# Transactivation of elements in the human endogenous retrovirus W family by viral infection

**DOI:** 10.1186/1742-4690-3-44

**Published:** 2006-07-06

**Authors:** Christoffer Nellåker, Yuanrong Yao, Lorraine Jones-Brando, François Mallet, Robert H Yolken, Håkan Karlsson

**Affiliations:** 1 The Department of Neuroscience, Karolinska Institutet, Retzius väg 8, 171 77 Stockholm, Sweden; 2 The Stanley Division of Developmental Neurovirology, The Johns Hopkins University School of Medicine, 600 N Wolfe Street, Blalock 1105, Baltimore, MD, 21287-4933, USA; 3 UMR 2714 CNRS-bioMérieux, IFR128 BioSciences Lyon-Gerland Ecole Normale Supérieure de Lyon, 46 allée d'Italie, 69364 Lyon cedex 07, France

## Abstract

**Background:**

Aberrant expression of human endogenous retrovirus (HERV) elements in the W family has previously been associated with schizophrenia, multiple sclerosis and preeclampsia. Little is know regarding the basal expression, transcriptional regulation and functional significance of individual HERV-elements. Since viral infections have previously been reported to transactivate retroviral long terminal repeat regions we examined the basal expression of HERV-W elements and following infections by influenza A/WSN/33 and Herpes simplex 1 viruses in human cell-lines.

**Methods:**

Relative levels of transcripts encoding HERV-W elements and cellular genes were analyzed by qPCR methods. An analysis of amplicon melting temperatures was used to detect variations in the frequencies of amplicons in discrete ranges of such melting temperatures. These frequency-distributions were taken as proxy markers for the repertoires of transcribed HERV-W elements in the cells.

**Results:**

We report cell-specific expression patterns of HERV-W elements during base-line conditions. Expressed elements include those with intact regulatory long terminal repeat regions (LTRs) as well as elements flanked by truncated LTRs. Subsets of HERV-W elements were transactivated by viral infection in the different cell-lines. Transcriptional activation of these elements, including that encoding syncytin, was dependent on viral replication and was not induced by antiviral responses. Serum deprivation of cells induced similar changes in the expression of HERV-W elements suggesting that the observed phenomena are, in part, an effect of cellular stress.

**Conclusion:**

We found that HERV-W elements, including elements lacking regulatory LTRs, are expressed in cell-specific patterns which can be modulated by environmental influences. This brings into light that mechanisms behind the regulation of expression of HERV-W elements are more complex than previously assumed and suggests biological functions of these transcripts.

## Background

Human endogenous retroviruses (HERV) are assumed to be remnants of ancient retroviral infections of our ancestors' germ-line cells. HERV sequences constitute approximately 3–8% of the human genome and can be classified into at least 31 families [1,2]. Tissue-specific hybridization patterns toarrays of sequences representative of different HERV families was recently reported, indicating a discrete and diversified regulation of their transcriptional activities [3,4].

The differential detection of *pol *transcripts related to one of these families, HERV-W [5], was previously observed in cerebrospinal fluids obtained from patients with multiple sclerosis [6] and patients experiencing their first manifestations of schizophrenia or schizoaffective disorder [7] as compared to control individuals. A recent study reported similar hybridization signals to a HERV-W *pol *sequence in prefrontal cortex samples from postmortem brains from patients with a long standing history of schizophrenia or bipolar disorder and control individuals [8].

According to Pavlicek *et.al. *[9] the human genome contains 654 HERV-W elements, the majority of which are comprised of long terminal repeat regions (LTR) lacking internal sequence. The remaining elements were classified into 2 major categories, a total of 77 retroelements with proviral structure containing intact LTRs and complete or partial internal sequences (*gag*, *pol *and *env *genes). In addition, 149 pseudoelements with internal sequences were found, lacking the regulatory U3 region of the 5'-LTR and the U5 region of the 3'-LTR. Structurally these copies resemble retroviral mRNAs and are thought to originate from LINE-mediated reverse transcription of such mRNAs. The remaining elements were grouped together in a third category based on lack of diagnostic regions due to truncations [9]. Due to the absence of regulatory promoter regions, these latter groups have been suggested to be non-transcribed [9,10]. However, except for a proviral element in ERVWE1 locus, which contains an intact *env *gene encoding syncytin [11], basal transcriptional activities of individual HERV-W elements remain poorly defined. Furthermore, potential regulation of individual HERV-W element expression is even less studied.

Herpes simplex viruses are known to transactivate retroviral regulatory LTR regions of both exogenous and endogenous human retroviruses, reviewed in [12]. With regard to HERV-W elements, induction of protein expression by HSV-1 was recently reported [13]. Although Influenza A virus has in one study been reported to transactivate the HIV-1 LTR [14], the influence RNA viruses on the transcriptional activities of HERV-W elements has, however, not been studied. Consequently we investigated the transcriptional activities of different HERV-W elements in human cell-lines during baseline conditions and how these are modulated by viral infections.

## Results

### Virus infection transactivates HERV-W elements

**Figure 1:**
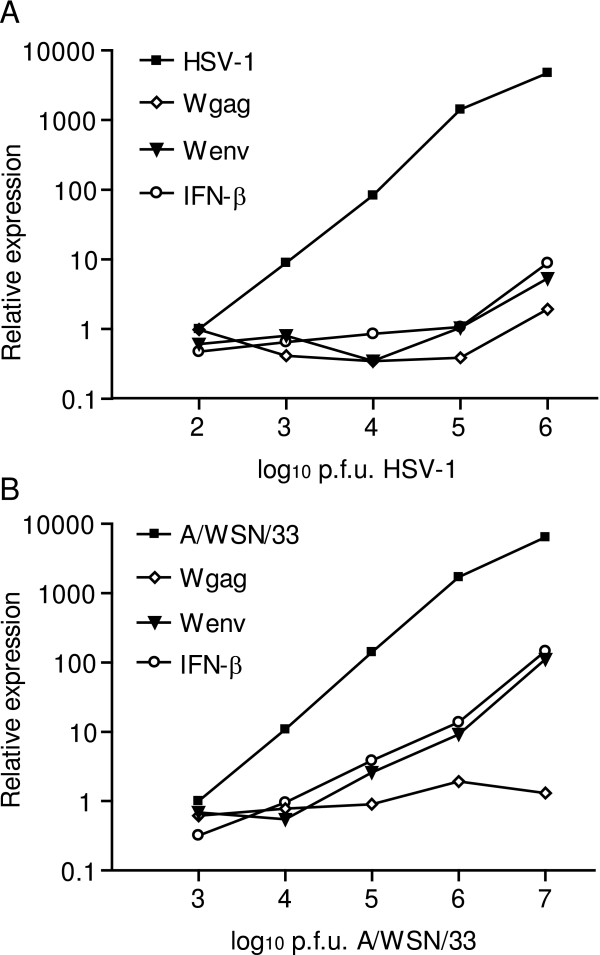
Gene expression in infected SK-N-MC cells. Relative levels of transcripts from HERV-W *env*, HERV-W *gag *and *IFNB1 *in SK-N-MC cells infected with increasing doses of herpes simplex type 1 (A) or influenza A/WSN/33 (B) as compared to uninfected control cell-cultures. Relative levels of transcripts from the *US6 *gene of herpes simplex type 1 (A) and segment 8 of the influenza A/WSN/33 virus strain (B) were determined in infected cultures. Relative levels of viral transcripts were normalized to those observed in cells infected with the lowest dose of each virus (n controls = 4, n virus = 5).

Initially, the neuroepithelioma cell-line (SK-N-MC) was infected with increasing titers of herpes simplex virus 1 (HSV-1) or influenza A/WSN/33 viruses. 24 or 48 hours post infection, the levels of transcripts from the latency gene 1 of HSV-1 and segment 8 of the influenza A/WSN/33 virus genomes were proportional to the respective numbers of viral plaque forming units (pfu) added to the cultures as determined by quantitative real-time PCR (Figure 1A and Figure 1B). Linear regression analyses revealed dose-dependent elevations of the relative levels of transcripts from HERV-W related *gag *and *env *genes elements 24 hours after infection with HSV-1 (Figure 1A). Cells infected with influenza A/WSN/33 virus responded with a dose-dependent increase in the relative levels of HERV-W *env*, but not *gag*, transcripts (Figure 1B). Reverse transcriptase controls were consistently found to be negative (data not shown). Variations in the levels of transcripts encoding IFN-β appeared to correlate with those from HERV-W *env *elements in response to both HSV-1 and influenza A/WSN/33 infections. By these experiments we were thus able to confirm the previous studies reporting induction of HERV-W by HSV-1 and make the novel observation that also influenza A/WSN/33 virus infection can transactivate HERV-W elements. In following experiments, 10^6 ^pfus of the influenza A/WSN/33 virus were used, corresponding to 0.5 multiplicity of infection (MOI).

**Figure 2:**
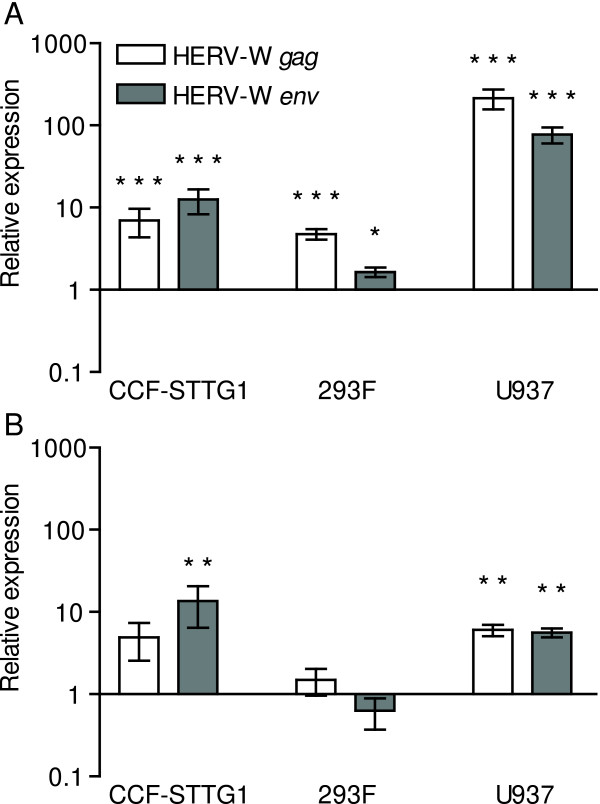
Expression of HERV-W elements in human cell-lines following influenza A/WSN/33 virus infection (A) or serum deprivation (B). CCF-STTG1, 293F and U937 cells infected with influenza A/WSN/33 virus (n = 7-9) were analyzed for HERV-W related transcripts relative to uninfected control cells (n = 7-12). Cells deprived of serum (n = 5-7) were analyzed for HERV-W related transcripts relative to control cells in serum enriched culture media (n = 5-8). Error bars indicate the standard error of the difference between the means of infected or serum deprived cells and corresponding control cells. Statistical significance is indicated by * = p < 0.05, ** = p < 0.01, *** = p < 0.001.

We next investigated if similar effects of the influenza A virus infection could be observed in other human cell-lines. The subsequent experiments were therefore conducted on the human astrocytoma cell-line, CCF-STTG1, the human histiocytic lymphoma cell-line U937, as well as 293F cells derived from human kidney. At baseline, all these cell-types contained detectable levels of transcripts from both HERV-W *gag *and *env *genes. Such transcripts were detected at significantly elevated levels in all three cell-types 24 hrs after infection (Figure 2A). U937 cells exhibited the highest relative increase in the levels of these transcripts.

### Relative increases in HERV-W element transcription after serum deprivation

Since influenza A viruses induce the expression of a variety of cytokine and pro-apoptotic genes in infected cells, we next investigated the levels of HERV-W transcripts in response to double-stranded RNA or replication-incompetent virus. CCF-STTG1 cultures were therefore exposed to poly(cytidylic-inosinic) acid (poly(I:C)) to activate protein kinase R (PKR) or heat-inactivated influenza A/WSN/33 virus to simulate effects of viral binding and fusion in the absence of viral replication. Poly(I:C)-treatment elicited a 33-fold increase in the levels of transcripts encoding IFN-β indicating adequate stimulation (data not shown). However, no significant alterations in the relative levels of HERV-W transcripts were observed in cells treated with poly(I:C) or heat-inactivated virus as compared to untreated controls (data not shown). In addition to mechanisms mediated by PKR, influenza A virus infections can induce apoptosis in infected cells by complex mechanisms not yet fully understood [15]. We next serum deprived the different cell-lines to induce stresses including cellular events leading to apoptosis [16-18]. CCF-STTG1 cells showed a small but significant increase only in the levels of *env *transcripts after serum deprivation. Serum deprived U937 cells exhibited significantly elevated levels of both HERV-W *gag *and *env *transcripts whereas the expression of these transcripts remained at baseline levels in 293F cells (Figure 2B). Thus, induction of HERV-W expression by influenza A/WSN/33 virus appears to be, at least partially, an effect of events leading to apoptosis during infection. Whether the relatively larger responses observed in infected cells as compared with serum deprived cells stems from additional actions of viral proteins or cellular responses to viral load remains undetermined.

### Qualitative analysis of HERV-W *env *and *gag *expression

**Figure 3:**
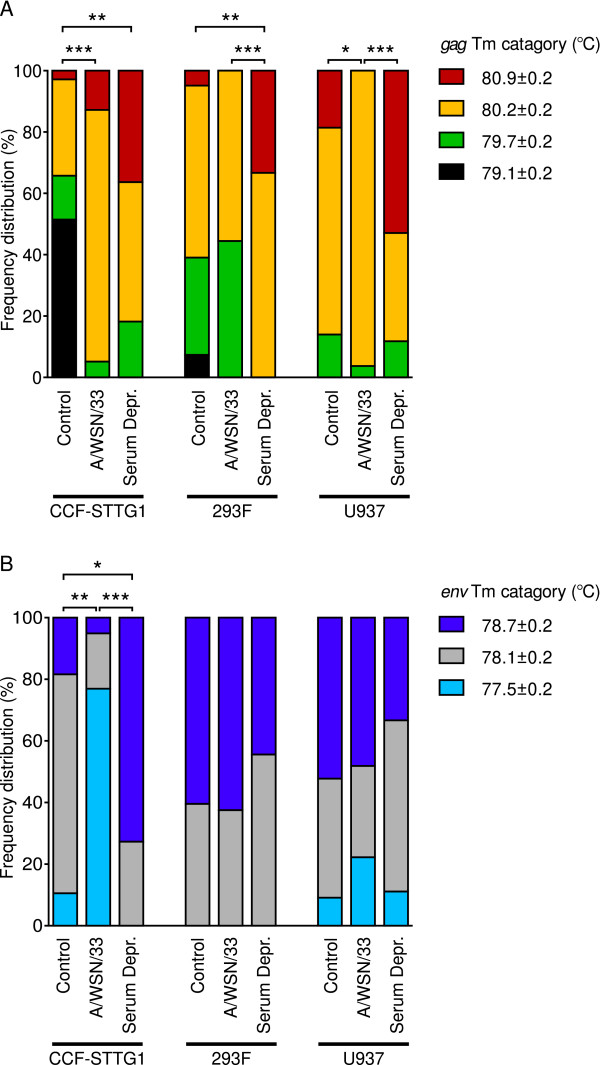
Influence of influenza A/WSN/33 virus infection and serum deprivation on the detectable frequency distribution of transcribed HERV-W related sequences in CCF-STTG1, 293F and U937 cells. (A) Distribution of detected HERV-W *gag *amplicons into four melting temperature ranges observed in control cells (n = 38-44), influenza A/WSN/33 infected cells (n = 24-39) and serum deprived cells (n = 11-18). (B) Distribution of detected HERV-W *env *amplicons into three melting temperature ranges observed. Statistical significance is indicated by * = p < 0.05, ** = p < 0.01, *** = p < 0.001.

We observed variations in Tm's of transcripts amplified in the assays for HERV-W *gag *and *env *in response to infection or serum deprivation and between controls of the different cell-lines. This was taken to be indicators of sequence variations and cloning and sequencing of amplicons from each of the different Tm categories supported this view (sequences and Tm's, Table 2). Sequenced products showed the greatest homology to previously identified HERV-W sequences in all cases as determined by BLAST  analyses. However, while each sequence was only detected in one Tm category, every Tm category encompassed multiple sequences. Thus, we conducted analyses of frequency distribution of amplicons into distinguishable HERV-W *gag *and *env *Tm categories (Figure 3A and 3B respectively) and compared these distributions between control, virus infected and serum deprived cells. The frequency distribution of HERV-W *gag *amplicons in four different temperature ranges differed significantly between control and influenza A/WSN/33 infected CCF-STTG1 and U937, but not 293F, cells (CCF-STTG1 χ^2 ^= 32.09, df = 3, p < 0.0001, U937 χ^2 ^= 8.523, df = 3, p = 0.0363). Differences were also observed between control and serum deprived CCF-STTG1 and 293F cells (CCF-STTG1 χ^2 ^= 14.86, df = 3, p = 0.0019, 293F χ^2 ^= 14.73, df = 3, p = 0.0021). Significant differences were observed between influenza A/WSN/33 infected and serum deprived 293F and U937 cells (293F χ^2 ^= 17.22, df = 3, p = 0.0006, U937 χ^2 ^= 20.63, df = 3, p < 0.0001). Frequency distributions of HERV-W *env *amplicons differed significantly between control, infected and starved CCF-STTG1 cells (controls and influenza A/WSN/33 infected samples χ^2 ^= 34.42, df = 2, p < 0.0001, controls and serum deprived cells χ^2 ^= 12.05, df = 2, p = 0.0024, influenza A/WSN/33 infected and serum deprived samples χ^2 ^= 28.44, df = 2, p < 0.0001). The Tm distributions of HERV-W *env *amplicons did, however, not differ between control, infected or serum deprived samples of either 293F or U937 cells.

To identify the specific HERV-W elements expressed, all sequences obtained were mapped to the human genome using Blat searches (Table 2). For those sequences without assignment to a unique genomic position, the numbers of homologous matches found are given in Table 2. For unambiguously mapped elements, 5000 upstream bases were subsequently downloaded and subjected to RepeatMasker analyses for the presence of HERV-W related 5'-LTR regions. According to these analyses, one transcribed element on chromosome 1q42 contains an intact HERV-W 5'-LTR whereas three transcribed elements on chromosomes 3q26, 12p13 and 15q21 all have partial HERV-W 5'-LTRs lacking the U3 region. An additional three transcribed elements on chromosomes 5p13, 12p12 and Xq22, however, lack discernible HERV-W 5'-LTRs.

### Transactivation of specific HERV-W elements by influenza A virus

**Figure 4:**
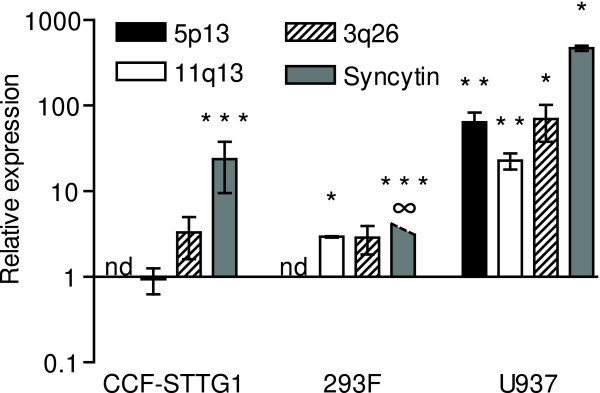
Expression of specific HERV-W elements following influenza A/WSN/33 infection. Levels of transcripts from the HERV-W *gag *on chromosomes 5p13, 11q13, 3q26 and the HERV-W *env *ORF encoding syncytin on 7q21 in CCF-STTG1, 293F and U937 cells infected with influenza A/WSN/33 (n = 3-7) relative to uninfected control cells (n = 3-9). Transcripts from 5p13 were not detectable in CCF-STTG1 or 293F cells in either control or infected cells, indicated by **nd **(not detectable). Syncytin transcripts were not detected in 293F control cells but were readily detectable (ct 34–35 using 500 ng input total RNA) in influenza A/WSN/33 infected cells, resulting in an infinite relative expression as indicated by ∞. Statistical significance is indicated by * = p < 0.05, ** = p < 0.01, *** = p < 0.001.

To determine if only proviral elements with intact 5'-LTRs or also elements with truncations or deletions in this region were modulated by infection, we specifically assayed the levels of transcripts from a HERV-W proviral element, two pseudoelements, as well as one element devoid of identifiable LTR. We chose to analyze the levels of transcripts from the aforementioned *env *gene on chromosome 7q21 which is part of a proviral element and encodes the full-length envelope protein, syncytin. Elements on chromosomes 3q26 and 11q13 were selected as examples of HERV-W pseudoelements. The element on 3q26 was unambiguously mapped and has a long ORF capable of encoding a putative matrix and carboxyterminal-truncated capsid proteins [19]. 11q13.5 was, in a different study, identified as differentially expressed in blood cells from recent onset schizophrenia patients (Yao et al, in preparation). Finally, the unambiguously mapped HERV-W element on 5p13 lacks identifiable LTR. Low levels of *env*-transcripts from the proviral HERV-W element at 7q21 were detected CCF-STTG1 and U937, but not 293F cells at base-line. Following infection, CCF-STTG1 and U937 cells displayed significantly elevated levels of these transcripts (Figure 4). *Env*-transcripts from this proviral element were also readily detectable in 293F cells following infection, thus indicated by the (∞) symbol in Figure 4. Transcripts from the HERV-W *gag *gene on 3q26 were detected in all cell types studied at baseline and the levels of these transcripts were significantly elevated (70-fold) in infected U937, but not in CCF-STTG1 or 293F cells. *Gag*-transcripts from the HERV-W pseudoelement on chromosome 11q13 were also present in all cell-types at baseline but were detected at significantly elevated levels in both 293F and U937 cells following infection. *Gag*-transcripts from the HERV-W element on 5p13, lacking upstream LTR could not be detected in CCF-STTG1 or 293F cells either at baseline or following infection. U937 cells, however, contained readily detectable levels of 5p13 *gag *transcripts at baseline and were significantly elevated (64-fold) following infection.

### EZ4U assaying for syncytin mediated cytotoxicity

The transcripts encoding syncytin, found to be transactivated in all cell types studied are normally only found in a cell population in the placenta. To resolve possible functional consequences of ectopic expression of this element CCF-STTG1 cells were transfected with an expression plasmid containing the full-length *env *gene from 7q21 encoding syncytin. These cells were subsequently assayed for mitochondrial function through the reduction of tetrazolium salts. 24 hours after transfection, 25% less tetrazolium salts were reduced in syncytin-transfected cells as compared to mock-transfected cells (Unpaired t test with Welch's correction P < 0.0001, data not shown). However, as compared to cells over-expressing enhanced GPF, syncytin expression caused a 12% decrease in cell proliferation/viability (Unpaired t test with Welch's correction P = 0.0150).

## Discussion

Through melting-temperature differences of amplicons generated using SYBR-Green chemistry followed by cloning and sequencing, we here report the constitutive expression of several different HERV-W elements in human cell-lines. Transcripts from genomic elements with complete, partial as well as absent 5'-LTRs were detected. Furthermore, we report that viral infections *in vitro *elevate the transcript levels from select HERV-W elements, including elements lacking HERV-W 5'-LTR regulatory regions.

Transcripts from elements in the HERV-W family have previously been detected by RT-PCR in most human organs as well as in different cell-lines of human origin [20]. Transcripts from HERV-W *pol *genes were recently reported to be present at high levels in the placenta, whole brain, adrenal glands and testis [21]. The relative contribution of the different HERV-W elements to the total levels of *pol *transcripts in different organs was not examined. The differences in the levels of transcripts from HERV-W *gag*, *pol *and *env *genes observed in different tissues and cell-lines might be attributed to the documented variations in promoter activities of U3-regions of HERV-W LTRs [22,23]. In addition, enhancer elements outside of the LTR can influence the transcriptional activities of HERV-W LTR promoters. This has been documented for the ERVWE1 locus on chromosome 7q21 which is regulated by promoter activity in the U3 region of the 5'-LTR as well as an upstream regulatory region [24]. Based on our present data there is constitutive, albeit low, expression of various HERV-W elements in human cell-lines. Moreover, the base-line relative transcript levels from different elements appeared to differ between these cell-lines. Sequencing of amplified products and mapping to genomic regions followed by RepeatMasker analysis indicated the presence of transcripts from HERV-W elements previously assumed to be transcriptionally silent due to truncations of the U3-region or complete lack of identifiable 5'-LTR [9,10]. The presence of such transcripts was subsequently verified by element-specific assays. We suggest that unidentified promoters, direct the expression of such HERV-W elements as has been described for cellular genes [25] in other studies. An analysis of eight different HERV-W *gag *transcripts previously identified in plasma samples from recent onset schizophrenia patients [26] revealed transcripts from one proviral element, six pseudoelements and one element lacking identifiable HERV-W 5'-LTR. Thus, elements lacking LTR regulatory regions appear to be transcribed *in vivo *and not only in cell-lines. In the present study, many of the transcribed HERV-W elements which could be mapped to single genomic sites were located in intronic regions of host genes. This is noteworthy as this is the case for only a minority of HERV elements in general [27]. These intronic HERV-W elements were all oriented opposite to the direction of the host gene transcription. Our findings illustrate the importance of detailed characterization of disease-associated transcripts in order to approach the mechanisms underlying their aberrant expression.

We here report that influenza A/WSN/33 can induce an elevation in the levels, to various degrees depending on host cell type, of transcripts related to HERV-W *gag *and *env*. The transactivating capacity of infectious agents on retroviral LTRs has previously been documented, e.g. HSV-1 has been reported to transactivate HIV-1 [12]. *In vitro*, the HIV-1 LTR has been reported to be stimulated also by influenza A virus [14]. Members of the *Herpesviridae *family can also activate LTRs of endogenous retroviruses including those related to HERV-W [28-30], which is also supported by our present study. Increased expression of HERV-W related envelope protein was also reported in response to HSV-1 but not rabies virus infection in neuroblastoma cells [31]. HSV-1 was recently reported to induce the expression of HERV-W gag protein expression [13].

We find that the mechanisms conferring transcriptional activation of HERV-W elements upon influenza A/WSN/33 virus infection were not related to the antiviral response of cells to either double-stranded RNA or to viral capsid binding and fusion. Induction of cellular stress responses through serum deprivation did however, to some extent, mimic the effects of virus infection in terms of transcription of HERV-W elements. The reported relative sensitivities to serum deprivation of the cell-lines is; U937, CCF-STTG1 and 293F in falling order [16-18]. This sensitivity appears to correlate with the relative increases in HERV-W element transcript levels. Interestingly, in serum deprived 293F cells, despite no discernible influence on the relative amount of HERV-W transcripts, alterations in the relative levels of the transcribed elements were detected. The differences observed in the expression patterns of *gag *or *env *transcripts between influenza A/WSN/33 infection and serum deprivation suggest that the virus has specific effects beyond those related to cellular stresses.

Specific analysis of transcripts from 7q21 showed that the proviral element was transactivated by influenza A/WSN/33 virus in all cell-lines tested. Surprisingly, in U937, virus infection elevated the levels of transcripts from elements lacking 5'-U3 regulatory regions. In the other cell types the degree of transactivation was less pronounced. Transcripts from the element lacking identifiable LTR were not even detectable in CCF-STTG1 or 293F cells at either baseline or following infection. Thus, when examined at the individual level, the transcriptional regulation of HERV-W elements is considerably more complex than can be revealed by studying only the promoter activities of HERV-W LTRs.

The proviral element on 7q21, found to be transactivated in all cell-lines studied, contains the only HERV-W gene known to have been "domesticated" into the human genome [32]. The product of this conserved gene is called syncytin in light of its fusogenic activity [11,33]. Expression of syncytin is normally largely restricted to the placenta, where it is proposed to contribute to the biogenesis of the syncytiotrophoblast layer. In the present study, ectopic expression of syncytin in an astrocytoma cell-line was associated with a lower activity of mitochondrial dehydrogenases as a measure of cytotoxicity. Although the mechanisms mediating this effect remain to be identified, our findings support possible negative influences of ectopically expressed syncytin in multiple sclerosis [34,35]

That viral insults induce expression of endogenous retroviral sequences raises questions as to the evolutionary origins of this effect. The observed HERV expression could constitute a cellular defense reaction, with syncytin and/or other envelope proteins acting as potential receptor blockers, preventing further spread of the virus in analogy to the protection it offers to Spleen Necrosis Virus infections [36]. This scenario could also be considered a case of a viral hijacking of envelope expression in order to utilize the immunosuppressive properties of the transmembrane region of syncytin (reviewed in [37]). This is further supported by the presence of syncytin at the fetomaternal interface, a region of immunological conflict between mother and foetus [33,38]. Thus, aberrant syncytin expression could promote immune-system evasion and viral spread, implied indirectly by the fact that syncytin expression is greatly enhanced by virus replication. However, the vast majority of HERV-W elements have no identifiable long ORFs. We speculate that functional consequences of the expression of such sequences should be sought at the level of non-coding RNA (for a review [39]).

The present study gives no evidence of a link between exogenous virus infection and aberrant expression of HERV-W elements in human disease. It does, however, raise some points of relevance for future studies regarding the expression of HERV-W elements;

i) Environmental stressors can modulate the transcriptional activities of certain HERV-W elements which could thereby be markers for such insults. ii) Disease specific frequency distribution patterns of different transcripts need not be reflected in levels of HERV-W transcripts and can be missed by generic methods. iii) Different cell-types exhibit specific quantitative and qualitative differences in the detectable patterns of transcribed HERV-W elements. Thus, transcripts detected in one tissue can differ from those detected in other tissues in response to a common insult. iv) Non-coding HERV-W elements are apparently transcribed which should merit studies into their transcriptional regulation and biological relevance.

## Methods

### Cell types

Human neuroepithelioma cells, SK-N-MC (HTB-10), were grown in Dulbecco's Modified Eagle Medium: Nutrient Mixture F-12 (D-MEM/F-12) supplemented with 4 mM L-glutamine, 15 mM HEPES and 12.5% FBS. Human astrocytoma cells, CCF-STTG1 (CRL-1718), and human histiocytic lymphoma cells, U-937 (CRL-1593.2), were grown in RPMI 1640 adjusted to contain 1,5 g/l NaHCO_3_, 4,5 g/l glucose, 10 mM HEPES and 1.0 mM Na-pyruvate. 293F cells, derived from human kidney, purchased from Invitrogen (Carlsbad, CA), were grown in D-MEM/F-12. All other cell-lines were obtained from the American Type Culture Collection, Manassas, VA. All cell culture media were supplemented with 10% FBS and penicillin/streptomycin (Invitrogen) unless otherwise specified.

### Virus infection of cells in culture

SK-N-MC cells, plated 18 hours prior to infection in 6-well tissue culture trays, were rinsed twice with Hank's Balanced Salt Solution w/o Ca^2+ ^or Mg^2+ ^and then duplicate wells were inoculated with MEM 4% FBS without (negative control) or with known amounts (as determined by standard plaque assays) of HSV-1 (10^2 ^- 10^6 ^plaque forming units (pfu) per well) or influenza A/WSN/33 virus (10^3 ^-10^7 ^pfu/well). The infections were allowed to proceed for 24 (HSV-1) or 48 hours (influenza A) at 37°C/5% CO_2 _before RNA isolation, see below.

CCF-STTG1, 293F and U937 cells were washed with one plating volume of MEM and inoculated with MEM containing influenza A/WSN/33 (0.5 MOI) [40]. After 1 hr at 37°C in 5% CO_2_, cells were washed thrice in one plating volume of MEM. Complete media was added and infections were allowed to proceed for 24 hrs in a humidified 5% CO_2 _incubator at 37°C before RNA isolation, see below.

### Heat-inactivated virus and poly(I:C) treatment

Influenza A/WSN/33 virus was inactivated by heating at 56°C for 90 minutes [41]. CCF-STTG1 cells were washed once in a plating volume of MEM before inoculation as described above with the exception that heat-inactivated virus was also added to the culture medium. Poly(I:C) (Sigma-Aldrich, St. Louis, MO) was dissolved in nuclease-free water (Ambion Inc., Austin, TX) at 10 mg/ml and added to CCF-STTG1 cells at a final concentration of 100 μg/ml [42].

### Serum deprivation

CCF-STTG1, 293F and U937 cells were cultured in 35 mm plates with normal growth medium. Cells were washed with one plating volume of their corresponding growth medium without FBS. Cells were subsequently allowed to incubate in another plating volume of serum deprived culture media for 24 hrs in a humidified 5% CO_2 _incubator at 37°C before RNA isolation, see below.

### RNA preparation and reverse transcription

RNA was isolated using the RNeasy Mini kit in accordance with instructions supplied by the manufacturer (Qiagen). RNA was quantified by spectrophotometric analysis. Oligo(dT)-primed cDNA was subsequently generated from 150–500 ng of DNaseI-treated RNA (as previously described [43]) using Superscript II reagents (Invitrogen) according to instructions from the manufacturer. Control reactions without the addition of reverse transcriptase were included.

### Real-time PCR and data analysis

1 μl cDNA templates, including the controls generated in the absence of reverse transcriptase, were added to triplicate 25 μl reaction mixtures using Platinum SYBR Green qPCR Supermix UDG (Invitrogen) or TaqMan Universal PCR Master Mix (Applied Biosystems, Foster City, CA) reagents. An ABI Prism 7000 real-time thermocycler (Applied Biosystems) was used for all assays. Oligonucleotides were designed using Primer Express (Applied Biosystems) and ordered from Invitrogen (SYBR Green assays) or Applied Biosystems (TaqMan assay). The sequences of the primers and probes with corresponding design templates are provided in Table 1. The efficiencies of the different assays ranged from 89–91% calculated as previously described [44]. Representative products of each assay were cloned and sequenced as previously described [44]. Threshold cycle (Ct) values from the exponential phase of the PCR amplification plot for each target transcript were normalized to those encoding β-actin. From these values, fold-differences in the levels of transcripts between the two groups were calculated according to the formula 2^-ΔΔCt ^[45]. The non-parametric Mann Whitney test was used to compare the levels of transcripts between cell treatments unless otherwise stated. The melting temperature (Tm) for each amplicon was determined in the ABIprism SDS software (Applied Biosystems) by recording the temperatures corresponding to the maximal rate of dissociation of double-stranded DNA [46]. Analysis was performed through the classification of Tm's into discrete temperature ranges that could reliably be distinguished between assays. Amplicons representative of each of the detected Tm's were cloned and sequenced. Five sequences obtained in the HERV-W *gag *assay could be mapped to a unique genomic position (Table 2A). Four *gag *sequences could not be mapped to an exact position due to multiple matches. Similarly, two of the four different W *env *sequences detected could be mapped to exact positions in the genome (Table 2B). TOPO-TA (Invitrogen) cloning was performed according to the manufacturer's instructions. Plasmids were sequenced at KIseq (Karolinska Institutet) and sequences were aligned with ClustalW . Mapping of transcribed elements was performed using the BLAT algorithm (, May 2004 assembly). 4000 bases upstream of uniquely mapped genomic elements were screened by RepeatMasker for the presence of HERV-W family LTRs .

**Table 1:** Targets, primer and probe sequences and GenBank accession numbers of sequences used for assay design.

Target	Polarity	Sequence (5'-3')	Acc. no.
β-actin	Sense	AACCGCGAGAAATCATGTTTG	AY582799
	Antisense	CAGAGGCGTACAGGGATAGCA	
HERV-W *env*	Sense	CCAATGCATCAGGTGGGTAAC	n/a
	Antisense	GAGGTACCACAGACAAAAAATATTCCT	
Syncytin	Sense	GTTAACTTTGTCTCTTCCAGAATCGA	NM_014590
	Antisense	CATCAGATCGTGGGCTAGCA	
Interferon-β	Sense	ACCTCCGAAACTGAAGATCTCCTA	NM_002176
	Antisense	TGCTGGTTGAAGAATGCTTGA	
11q13 *gag*	Sense	GTTTGCGGCACCAATCTGT	n/a
	Antisense	CGATCTCTGGTATCTCAGGTCAATG	
HSV-1 glycoprotein D	Sense	TTTGCGGAATTGTGTACTGGAT	AY155225
	Antisense	GAGGCGTATGCGCTTTGG	
5p13 *gag*	Sense	CCTGAGGGCCATGACTAAAGAG	n/a
	Antisense	CCGCCTTAGGCCCAGAGT	
Seg. 8 A/WSN/33	Sense	CAGCACTCTCGGTCTGGACAT	U13683
	Antisense	TCCTTCAGAATCCGCTCCACTA	
HERV-W *gag*	Sense	TCAGGTCAACAATAGGATGACAACA	n/a
	Antisense	CAATGAGGGTCTACACTGGGAACT	
HERV-W 3q26.32 *gag *ORF, MGB-probe		6-FAM-CCTGTGGGAGTTGTT-MGB	AF156961

**Table 2:** Sequences and genomic positions of mapped HERV-W *gag *(A) and *env *(B) elements. Dashes indicate nucleotides that are identical with the prototypical HERV-W sequences. Open circles indicate gaps in sequence. Sequences that could not be unambiguously mapped to one genomic loci are indicated by the number of indistinguishable genomic loci found.

**A**			
**5' LTR**	**5' HERV-W *gag * 3'**	**Tm(°C)**	**Genomic location (strand)**

Truncated	GAGGAAooAGAACAACTCCCooACAGGCCAGCAGGC	80.2 ± 0.2	3q26:180256375-180256406(+)
None	--A---AG-------T----C---------------	80.2 ± 0.2	5p13:31432195-31432224(-)
Truncated	---------------T----C--o------------	80.9 ± 0.2	12p13:8809216-8809247(-)
None	------AG-------T----C------------A--	79.7 ± 0.2	12p12:18113623-18113657(+)
Complete	------AG-------T----CC--------------	80.2 ± 0.2	1q42:224124801-224124836(-)
n/a	--------------------C---------------	80.2 ± 0.2	2 elements
n/a	------AG-------T----C---------------	80.2 ± 0.2	15 elements
n/a	------AG-----G-T----C---------------	80.2 ± 0.2	11 elements
n/a	---------------T----C---------------	79.7 ± 0.2	4 elements

**B**			

**5' LTR**	**5' HERV-W *env* 3'**	**Tm(°C)**	**Genomic location (strand)**

n/a	TCCTCCCACACAAATAGTCTGCCTACCCTC	77.6 ± 0.2	5 elements
Truncated	---C---o---G------------------	78.1 ± 0.2	15q21:53385581-53385607(-)
None	A----------G------------------	78.7 ± 0.2	Xq22:106102713-106102742(-)
n/a	-----------G------------------	77.6 ± 0.2	5 elements

### Mitochondrial viability in response to syncytin expression

CCF-STTG1 cells were transfected with the plasmid PH74 [5] containing the full length ORF encoding syncytin using Lipofectamine 2000 reagents in accordance with the manufacturer's instructions (Invitrogen). Transfection with the pEBFP expression plasmid (Clontech, Mountain View, CA) encoding a variant of green fluorescent protein was used as a control for the effects of forced protein expression. CCF-STTG1 cells were transfected with expression plasmids at 70% confluence in 96-well plates. After 24 hours of incubation at 37°C and 5% CO_2_, toxicity was determined using the EZ4U kit [47] according to the manufacturer's instructions (Biomedica Medizinprodukte GmbH & Co KG, Wien).

## Competing interests

The author(s) declare that they have no competing interests.

## Authors' contributions

YY carried out melting temperature of amplicon analyses and critical review of the manuscript. LJ-B carried out the infections with influenza A/WSN/33 and HSV-1 virus. RHY participated in the design of the study. FM critically revised the manuscript. HK conceived the study, its design, coordinated it and drafted the manuscript. CN designed, carried out the cell culture studies, qPCR, statistical analyses and drafted the manuscript.
